# A VISION FOR THE FUTURE OF ASSISTED LIVING RESEARCH: LEVERAGING ADMINISTRATIVE DATA TO STUDY QUALITY

**DOI:** 10.1093/geroni/igac059.1118

**Published:** 2022-12-20

**Authors:** Kali Thomas, Paula Carder, Lindsey Smith, Sheryl Zimmerman

**Affiliations:** Brown University, Providence, Rhode Island, United States; Portland State University, Portland, Oregon, United States; Brown University, Providence, Rhode Island, United States; University of North Carolina, Chapel Hill, North Carolina, United States

## Abstract

Because the assisted living industry is neither federally regulated nor federally licensed, methodologies to study the quality of long-term care used in other settings (e.g., annual inspection reports, resident assessment data) do not exist for the large and growing number of assisted living communities and their residents. The objective of this presentation is to present a vision for the future of assisted living research to inform quality using large, national datasets. To do this, the presenter will discuss existing approaches to identify and characterize assisted living communities and their residents using administrative data. A specific focus will be to highlight the opportunities and challenges associated with each approach. The presenter will offer suggestions for possible enhancements in existing approaches and next steps in the study of assisted living aimed at understanding and improving the quality of care delivered in these settings.

